# The epidemiological transition in Antananarivo, Madagascar: an assessment based on death registers (1900–2012)

**DOI:** 10.3402/gha.v7.23237

**Published:** 2014-05-15

**Authors:** Bruno Masquelier, Dominique Waltisperger, Osée Ralijaona, Gilles Pison, Arsène Ravélo

**Affiliations:** 1 Centre de recherches en démographie et sociétés, Université catholique de Louvain (UCL), Louvain-la-Neuve, Belgium; 2 Institut National d’Etudes Démographiques (INED), Paris, France; 3 Health Statistics Division, Ministry of Health, Antananarivo, Madagascar; 4 Office of demography and social statistics, Institut National de la Statistique de Madagascar (INSTAT), Antananarivo, Madagascar

**Keywords:** epidemiological transition, Antananarivo, death registers, mortality, Madagascar, vital statistics, Africa

## Abstract

**Background:**

Madagascar today has one of the highest life expectancies in sub-Saharan Africa, despite being among the poorest countries in the continent. There are relatively few detailed accounts of the epidemiological transition in this country due to the lack of a comprehensive death registration system at the national level. However, in Madagascar’s capital city, death registration was established around the start of the 20th century and is now considered virtually complete.

**Objective:**

We provide an overview of trends in all-cause and cause-specific mortality in Antananarivo to document the timing and pace of the mortality decline and the changes in the cause-of-death structure.

**Design:**

Death registers covering the period 1976–2012 were digitized and the population at risk of dying was estimated from available censuses and surveys. Trends for the period 1900–1976 were partly reconstructed from published sources.

**Results:**

The crude death rate stagnated around 30‰ until the 1940s in Antananarivo. Mortality declined rapidly after the World War II and then resurged again in the 1980s as a result of the re-emergence of malaria and the collapse of Madagascar’s economy. Over the past 30 years, impressive gains in life expectancy have been registered thanks to the unabated decline in child mortality, despite political instability, a lasting economic crisis and the persistence of high rates of chronic malnutrition. Progress in adult survival has been more modest because reductions in infectious diseases and diseases of the respiratory system have been partly offset by increases in cardiovascular diseases, neoplasms, and other diseases, particularly at age 50 years and over.

**Conclusions:**

The transition in Antananarivo has been protracted and largely dependent on anti-microbial and anti-parasitic medicine. The capital city now faces a double burden of communicable and non-communicable diseases. The ongoing registration of deaths in the capital generates a unique database to evaluate the performance of the health system and measure intervention impacts.

The theoretical model of the epidemiological transition describes a long-term shift from a regime of high and fluctuant mortality, dominated by infectious diseases, to a regime of low mortality where deaths are predominantly due to non-communicable diseases (NCDs) linked with population aging and changes in lifestyles (such as increasing fat and calorie consumption, decreasing exercise, and tobacco use) (1). In western countries, there has been considerable debate over the chronology of this transition and the contribution of better nutrition, rises in living standards and advances in medical science to the decline in mortality (2, 3). The relevance of this model for Latin America and Asia has also been widely debated (4–7). Less attention has been devoted to sub-Saharan Africa (SSA), apart from a few case studies on Accra (8), South Africa (9), and Mauritius (10). In SSA, the epidemiological transition started only after the World War II, largely thanks to public health measures that were internationally supported (11). It has progressed at a much slower pace than in other developing regions. For instance, although life expectancy was around 36 years in SSA and India in 1950–1955, it had reached 65 years in India in 2005–2010, against only 53 in SSA (12). Over the past 30–40 years, child mortality has declined dramatically in most African countries, but conspicuous stalls were observed in the 1990s and early 2000s. These setbacks were related to the HIV epidemic, temporary declines in immunization rates and upsurges in malaria-related mortality (13). Declines in child mortality have not been matched by sustained improvements in adult survival either. Since the 1990s, most countries in eastern and southern Africa have witnessed enormous surges in adult mortality because of AIDS. Progress in adult survival has been limited even in countries whose HIV epidemics have been small. Madagascar is an exception in this regard, because adult and child mortality rates have evolved in concert toward a regime of low mortality, as they have in Ethiopia or Senegal. According to estimates from the Demographic and Health Surveys (DHS), the probability that a newborn would die before reaching age 5 (_5_q_0_) in Madagascar was 0.20 in 1985 and had declined to 0.08 by 2005. The probability that a person aged 15 would die before reaching age 60 (_45_q_15_) was around 0.36 in 1985 (among males) and had declined to 0.28 by 2005 (13). As a result, no country in SSA had a higher life expectancy than Madagascar during the period 2005–2010 (62 years), except for small islands such as Cape Verde and Mauritius (12). And yet Madagascar is among the poorest countries in the region, with 93% of the population living on less than $2 a day (14).

This disconnection between health and economic progress calls for a detailed examination of the epidemiological transition occurring in Madagascar. To date, this has been hindered by the lack of long-term series of data on mortality by cause, due to the low coverage of death registration, estimated at less than 50% at the national level (15). Although this low coverage is a feature of most countries in SSA, the vital registration system is atypical in Madagascar because it was established relatively early, in 1878, by the Merina Kingdom, which ruled over most of the island at that time. The system only covered areas controlled by this Kingdom, however, and also did not include the slave population. The French colonial administration officially abolished slavery in 1896 and enforced vital registration throughout the island. Vital registration offices were set up in all districts, and Antananarivo, the capital city, was considered an administrative unit on its own, with its own offices (16). Since then, the coverage of death registers has always been very high in the city, in contrast to other parts of the country. In this paper, we take advantage of this wealth of data and describe the epidemiological transition that occurred in city over the period 1900–2012. We first provide some historical background and introduce our data sources and our methods, and then we explore the changes in patterns of mortality by age groups and cause.

## Historical background

Antananarivo, formerly known as Tananarive, is located in the central highlands of Madagascar at an altitude of 1,250–1,470 m. It is spread across 90 km^2^. Its climate is subtropical with a cold and dry season from May to October (with an average minimum temperature of around 10°C) and a hot and rainy season from November to April (with an average maximum temperature of around 27°C).

At the end of the 19th century, the mortality regime of the city was characterized by high death rates from malaria (causing about 25% of deaths in 1895), smallpox, syphilis, and tuberculosis (17). Epidemics were frequent; Antananarivo was hit by smallpox epidemics in 1875–1881 and 1884–1889, a flu epidemic in 1890 and a typhoid epidemic in 1894 (17, 18). The incidence of infectious diseases apparently increased when the Merina Kingdom reinforced its coercive policies from the 1870s. Massive movements of units of forced labor contributed to the spread of malaria over the central highlands in 1878, in conjunction with the resettlement in the valleys of populations that previously lived in fortified centers located over the hills.

After the French takeover, the colonial administration acted quickly to implement measures to increase the health of the indigenous workforce and promote population growth. By 1896, the city had a hospital and a medical school to train local doctors and midwives. The Indigenous Medical Assistance was established the following year and provided health care to the population free of charge. A Pasteur Institute was founded in 1899 to produce locally the treatment for rabies and the smallpox vaccine. Routine immunization campaigns were organized and smallpox was considered well controlled in 1908.

Unfortunately, these measures were partly offset by the expansion of malaria. This is apparent in the mortality data routinely collected in Antananarivo that we describe below. In February 1906, during the rainy season, 57% of deaths were due to malaria, against only 10% in February 1903; it went back down to 35% in February 1907. This expansion of malaria was linked to a broad set of factors, including large movements of population induced by infrastructure projects, changes in the methods used for rice cultivation (rice plants were no longer dried out after the harvest), and the settlement of some populations closer to the rice fields (19). The French administration established quinine depots, but malaria nonetheless became endemic in the central highlands.

The colonial administration also developed the city by clearing some marshland, setting up a public lighting system in 1910 and installing the first standpipes in 1911 (20). In 1916, a Municipal Office of Hygiene (*Bureau municipal d’hygiène –* BMH) was created. The BMH worked under the supervision of the mayor to deliver free consultations, conduct immunization campaigns, provide malaria prophylaxis, and isolate patients affected by highly infectious diseases such as the plague. The plague reached Antananarivo in 1921, and up to 140 deaths were reported in 1927 (when the city had 73,000 inhabitants). The BMH responded to this outbreak with very drastic measures including isolation of infected patients into plague houses and the disinfection or destruction by fire of the patients’ homes. The introduction of sulfonamides and streptomycin helped reduce the incidence of the plague and no human cases were notified between 1949 and 1978, when it reemerged in the city.

Penicillin and other antibiotics were introduced in Antananarivo in the late 1940s and early 1950s, but little is known on the timing of their dissemination and their use in medical facilities. The importance of chloroquine in the fight against malaria is more documented (21). Beginning in 1949, the BMH launched a massive anti-malaria program, based on the indoor spraying of DDT and chemoprophylaxis administered on a weekly basis to school-age children and preschoolers. Malaria was considered under control in the central highlands in 1960, when Madagascar gained its independence, and the DDT spraying was ceased. The Expanded Program of Immunization (EPI) was launched in 1976, starting with diphtheria, tetanus, pertussis, and tuberculosis. The polio vaccine was included in the immunization schedule in 1982 and the measles vaccine in 1985.

**Fig. 1:**
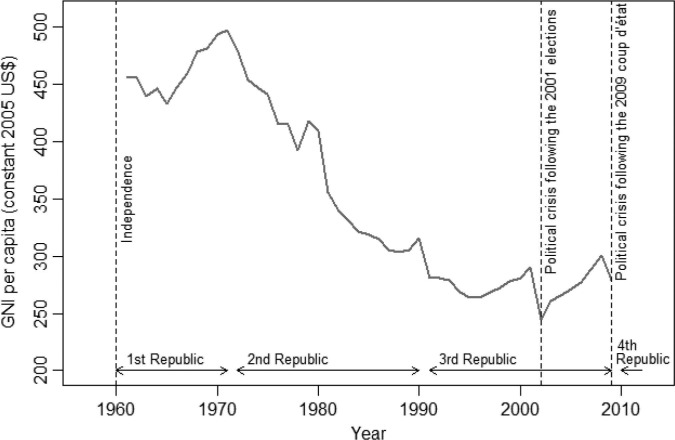
Trends in GNI per capita (1961–2009), according to data from the World Bank (constant 2005 US$) (14).

The beneficial effects of these programs were stymied by a major economic crisis that began as early as 1972. This year marked the end of Madagascar’s First Republic (1960–1972); the government was overthrown by a popular revolt after growing criticism for maintaining too strong ties with France. GNI per capita then began a steep decline that lasted until the mid-1990s (Fig. 1); it dropped from $497 in 1971 to $264 in 1996 (14). The shift toward economic insularity under the socialist-Marxist regime of Didier Ratsiraka (1975–1993) contributed to this disastrous situation. The regime took control over the agricultural production and marketing system and nationalized foreign-owned trading and industrial companies. The import trade was also put under state control. The price paid to rice producers fell and many smallholders withdrew into subsistence farming. In 1977, rice had to be imported to feed the population, despite Madagascar having been a net exporter of rice 5 years earlier. In 1981, the country removed subsidies on rice and started to implement a series of structural adjustment programs. Rice imports remained controlled by the state until 1986, even though controls over the prices paid by the consumers had been lifted. A parallel market blossomed, where rice was sold at about twice the price of the state-controlled market (22).

To make things worse, malaria resurged in Antananarivo in 1984, at a time when many treatment and prophylaxis centers had closed after their funding was cutoff by the central administration (23). The resumption of chemoprophylaxis with chloroquine in 1988 and the reintroduction of indoor DDT spraying in 1993–95 brought malaria back under control. Today, cases of autochthonous malaria are very rare in Antananarivo, but seroprevalence remains high because inhabitants come into contact with the parasite while travelling outside the city (24).

Madagascar was forced to introduce a series of structural reforms and move away from state intervention in the late 1980s. Didier Ratsiraka lost the elections in 1993 but he was voted back into power in 1997 and he moved further toward economic and financial liberalization. The economic recovery was short lived as a controversy over the 2001 presidential election gave rise to violent clashes in 2002 and resulted in a 6-month standoff. GNI per capita plunged, rose again but dropped in 2009 when the president Ravalomanana was ousted from power during a coup (Fig. 1). Restrictions on aid from foreign governments and other donors, which resulted in severe cuts in public spending, followed this unconstitutional change of power. A presidential election was organized in 2013 to put an end to Madagascar’s political impasse.

The economic decline and the recurrent political instability have not prevented Madagascar from achieving significant health improvements. Empirical evidence from four DHS surveys indicate both the substantial decline in under-five mortality in Madagascar over the past three decades, and a consistent advantage of Antananarivo over rural areas and other cities (Table 1) (25–28). This urban advantage has been observed in several African countries from the 19th century, because of higher incomes, higher education levels, and a disproportionate provision of modern sanitation and health care (29, 30).

**Table 1:** Under-five mortality rates for the 10-year period preceding each DHS survey conducted in Madagascar, according to type of place of residence. Sc: (25–28)

	1992 DHS	1997 DHS	2003/04 DHS	2008/09 DHS
Antananarivo-city	152	110	43	51
Other cities	137	132	81	70
Rural areas	183	174	120	84

In the case of Antananarivo, the lower mortality rates can be attributed to a variety of advantages. The capital has benefitted from a favorable location with a lower prevalence of malaria than in endemic coastal areas, although the region has been prone to epidemics. The city is also responsible for approximately 40% of Madagascar’s GNI and a higher proportion of households have access to basic amenities than in the rest of the country. As an example, the proportion of the population using an improved drinking-water source was 98% in the capital in 2008–2009 against only 32% in rural areas and 82% in the other cities (28). In the capital, 82% of households had access to electricity against 8% in rural areas and 63% in other cities. The city is better equipped with health facilities compared to the rest of the country; it has three university teaching hospitals that correspond to the highest level of the health delivery system. In addition, large differences are found in educational attainment: above age 6, only 3% of women do not have any education in the capital, compared with 20% in Madagascar (28). These differences are a remnant of the early establishment of the school system in the central highlands by the Merina Kingdom. Possibly 7% of the adult population of the region were literate in the mid-19th century (31). In 1960, 16% of the inhabitants of Antananarivo aged 15 and over had already attended school beyond primary level (32); this proportion rose to 64% in 1995 and to 71% in 2008/2009 (28).

## Materials and methods

Our assessment of the epidemiological transition in Antananarivo is based on three types of data sources: 1) monthly reports of deaths by cause for the period 1900–1907, 2) some published estimates for the period 1931–1951, and 3) individual-level data from death registers for the period 1976–2012.

First, monthly reports of deaths by cause started to be published in 1900 by the municipal administration in the *Journal Officiel de Madagascar et dépendances* (33). We accessed the reports for the period 1900–1907. They provide a breakdown of deaths by age, sex and cause, but without cross-tabulation. From this first type of data source, we will only extract the ranking of categories of causes of deaths and estimates of the crude mortality rate (CDR).

Second, starting in 1916, the BMH took over responsibility for the registration of deaths in the city and for regular reporting on mortality. Some published estimates of the CDR are available for the years 1931–1951 (21).

Third, all death registers maintained at the BMH for the period 1976–2012 were digitized, that is, about 298,000 records. When deaths occur at home (about 60% of deaths since 1976), relatives or caretakers of the deceased contact the BMH, and a physician is sent to the house to assign a cause of death, based on available medical documents and on post-mortem interviews with the family concerning the symptoms and circumstances preceding the death. For deaths occurring in hospitals or clinics, the reports are filled in by the medical personnel and transmitted to the BMH by the relatives. Since 1976, more than 80% of deaths have been reported on the day of death or the day after. A high coverage of deaths is maintained because cemeteries are guarded and reporting the death at the BMH is required to obtain a burial permit or to move the corpse. Based on data for the period 1984–1995, Waltisperger et al. showed that age-specific mortality rates based on the BMH conformed to standard age patterns of mortality, and concluded that the data presented no indication of underreporting of deaths (34).

For this analysis, we retained only the deaths of residents of Antananarivo. Causes of death were recorded according to the Ninth Revision of International Statistical Classification of Diseases (ICD-9). The same physician was in charge of recoding all deaths that had not been previously coded in hospitals or clinics. Causes were later consolidated into seven broad categories: 1) infectious diseases (classified in the first chapter of the ICD-9), 2) neoplasms, 3) nutritional deficiencies, 4) cardiovascular diseases (CVDs), 5) diseases of the respiratory system (including influenza and bronchitis), 6) injuries and accidents, and 7) other diseases. The complete list of corresponding ICD-9 codes is provided elsewhere (22). Since 1976, the percentage of deaths whose cause was unknown or ill-defined (including senility) has ranged from 7.5 to 18%, the maximum having been attained in 1988, shortly after a mortality crisis that led to a higher-than-average proportion of deaths occurring at home. The percentage of unknown or ill-defined causes then declined to below 10%, but it has risen again in recent years due to the increasing share of deaths occurring at ages 70 and above. For the present analysis, deaths of unknown or ill-defined cause were redistributed by age group and sex among the other categories of causes, assuming that imprecise statements concern all causes in the same proportion.

To estimate population exposure, we used a logistic curve to interpolate between population counts from administrative censuses taken annually from 1901 to 1963 (16, 35), counts from the 1975 and 1993 national censuses, and the provisional count that was done in 2009 as part of the mapping of the forthcoming census (which has been delayed because of the political crisis). Our estimates refer to the ‘Commune urbaine d’Antananarivo Renivohitra’ (CUA), corresponding to the six districts for which we have data from the BMH. Antananarivo had 53,600 inhabitants in 1901. The population increased rapidly from 1925 to attain 200,000 inhabitants around 1957. Over the past 60 years, it has increased more than five-fold, reaching 1.03 million in 2009. Yet its annual growth rate has been relatively modest in recent years (2.4% over the period 1975–2009), compared to other African capitals such as Kinshasa (5% over the period 1975–2010) or Ouagadougou (7%). Madagascar remains weakly urbanized and migration does not play a key role in shaping its capital. In 1995, only 27% of the residents of the city were migrants, and half of them came from the province of Antananarivo (32).

In order to compute age-specific mortality rates from registered deaths for the period 1976–2012, we had to distribute this population by age group and sex, which is challenging because the last two censuses carried out were in 1975 and 1993. Our strategy was to compute by sex the share of each age group in the population enumerated in the these two censuses, and in four DHS, conducted in 1992, 1997, 2003–2004 and 2008–2009, after selecting only the households that had been sampled in the city (25–28). We used penalized smoothing splines to interpolate the share of each age group between the surveys and the censuses (and extrapolated to 2012), and multiplied these percentages by the total population, estimated as explained above, to obtain a population count by age group, sex, and calendar year.

The level of detail of the mortality data thus varies over time. Age- and cause-specific mortality rates can only be computed from 1976 onwards, but summary indicators can be obtained from 1900. In the next section, we first present trends in the CDR in Antananarivo for the period 1901–2012 alongside trends in the CDR obtained from incomplete death registration for Madagascar from 1906 to 1972 (36–38). Second, using the monthly reports published in the beginning of the 20th century and the individual-level data from 1976, we compare the rankings of the leading causes of deaths, for both sexes combined. Third, we provide estimates of the life expectancy at birth by sex and under-five mortality rates from 1976. Finally, we detail changes in the standardized mortality rates by causes (1976–2012) and estimate the contribution of age groups and causes of deaths to changes in life expectancy, based on the algorithm developed by Andreev et al. (39).

## Results

### Trends in the crude mortality rate (1901–2012)

**Fig. 2:**
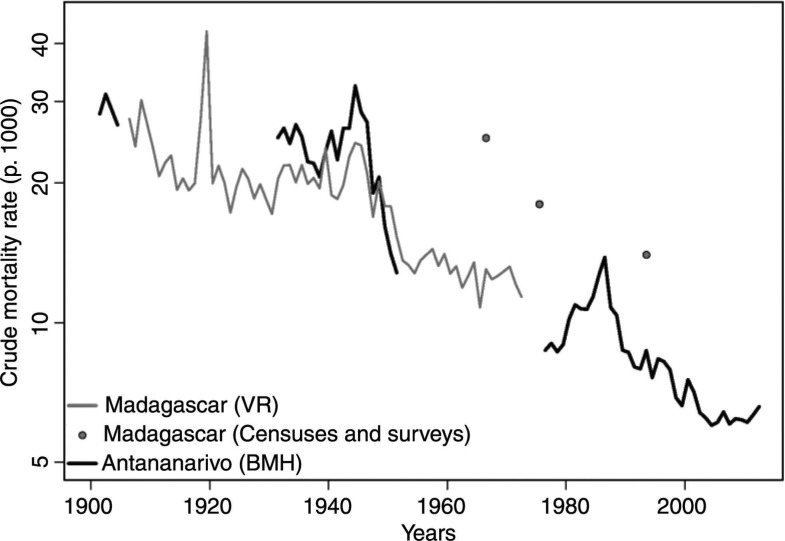
Trends in the crude death rate for Madagascar and Antananarivo city (1901–2012) (logarithmic scale). Sources: Refs. (21, 36–38) and data from the BMH for the period 1976–2012.

Caution is obviously required in interpreting long-term trends in CDR, because they can be distorted by changes in the age structure of the population over the period and variations in the completeness of death reporting. Based on the age distribution of registered deaths in 1965–67, Courbage and Fargues (40) estimated that about half of the male deaths and over one-third of the female deaths were not registered at the national scale. According to the demographic survey conducted in 1966, the CDR was around 25‰ at that time (41), much higher than the estimate based on vital registration (13‰ in 1966). Despite this massive underreporting of deaths, we can venture to say that the CDR was at least 30‰ at the start of the 20th century in Madagascar, and could have slightly declined until 1918, when mortality peaked above 40‰ following an outbreak of the Spanish flu (Fig. 2). It remained stable between 1920 and 1948, and declined sharply only after the World War II. The apparent stall between 1955 and 1972 might reflect an increase in the completeness of death reporting, because the 1975 census estimated the CDR at 18‰, compared with 25‰ in the 1966 survey. According to the 1993 census, the CDR had further declined to 14‰ by the beginning of the 1990s.

In Antananarivo, estimates from death registration have always been considered much more reliable than those based on nationwide data. In 1901, the CDR was estimated at 28.2‰ in the city (16). Little progress was registered prior to the end of the World War II; the CDR was still around 25‰ in 1931. It reached 32‰ in 1944 and then dropped to 13‰ in 1951 (21). This decline continued until the end of the 1970s, with the CDR reaching 8.8‰ during the period 1976–1979. From 1980, it rose again and peaked at 13.8‰ in 1986, when malaria resurged in the central highlands and increases in the price of rice resulted in acute food shortages. The CDR went down to 8.7‰ in 1989 and reached its lowest level in 2004 (6‰).

### Changes in the rankings of the leading causes of deaths (1900–2012)

To investigate changes in the cause-of-death structure, we distinguish three periods: 1900–1903 (the first uninterrupted series of monthly mortality reports by cause), 1976–1980 (the first 5 years covered by the database of individual death records), and 2008–2012 (the most recent years). Because the classifications of diseases changed between 1900 and 1976, as we discuss below, we will only examine the rankings of broad categories of causes of deaths. Problems remain, however, because some categories of causes such as nutritional deficiencies (corresponding to ICD-9 codes 260–269) were not singled out in 1900–1903. Measles was not mentioned among causes of deaths either, whereas it accounted for about 4% of deaths in 1976–1980. Despite this, this comparison reveals three general features (Table 2). First, there was a stark decline in the contribution of diseases of the respiratory system (pneumonia in particular), from 38% in 1900–1903 to 12.5% in 1976–1980 (and 10% in the last period). Second, apart from pneumonia, infectious and parasitic diseases continued to occupy a major position in the cause-of-death profile in 1976–1980. If all deaths from nutritional deficiencies had been reallocated to infectious causes, the proportion of deaths due to infections would have only declined from 34% in 1900–1903 to 29% in 1976–1980. This proportion plunged to 11.9% in 2008–2012. Most of the decline that happened before 1980 was due to reductions in malaria- and tuberculosis-related mortality, with intestinal infections still accounting for about 15% of all deaths in 1976–1980. Third, CVDs, neoplasms and trauma gained in importance, accounting for 32% of deaths in 1976–1980 and 51% in 2008–2012, against only 9% at the start of the 20th century.

**Table 2:** Rankings of the leading causes of deaths in 1900–1903, 1976–1980, and 2008–2012 in Antananarivo

Rank	Causes of death (1900–1903, all ages and both sexes)	Prop. (%)	Rank	Causes of death (1976–1980, all ages and both sexes)	Prop. (%)	Rank	Causes of death (2008–2012, all ages and both sexes)	Prop. (%)
1	(Broncho)-pneumonia, influenza, ARI	30.6	1	Cardiovascular diseases	21.6	1	Cardiovascular diseases	35.8
2	Intestinal infections	15.9	2	Intestinal infections	14.8	2	Other causes	10.6
3	Malaria	7.9	3	Other causes	10.5	3	Neoplasms	8.5
4	Tuberculosis	7.5	4	(Broncho)-pneumonia, influenza, ARI	9.6	4	Injury and accidents	6.7
5	Other diseases of respiratory system (including bronchitis)	7.2	5	Other infectious and parasitic diseases (incl. measles)	5.6	5	Diseases of the digestive system	5.5
6	Other causes	6.7	6	Neoplasms	5.5	6	Other diseases of respiratory system (including bronchitis)	5.2
7	Congenital anomalies and other perinatal conditions	6.5	7	Nutritional deficiencies	5.1	7	(Broncho)-pneumonia, influenza, ARI	4.8
8	Cardiovascular diseases	6.5	8	Injury and accidents	5.0	8	Congenital anomalies and other perinatal conditions	4.3
9	Whooping cough	2.1	9	Diseases of the digestive system	5.0	9	Tuberculosis	4.1
10	Diseases of genitourinary organs	1.9	10	Congenital anomalies and other perinatal conditions	4.5	10	Diseases of genitourinary organs	3.3
11	Neoplasms	1.9	11	Diseases of genitourinary organs	3.0	11	Diseases of the nervous system	2.8
12	Diseases of the nervous system	1.8	12	Other diseases of respiratory system (including bronchitis)	2.9	12	Other infectious and parasitic diseases (incl. measles)	2.6
13	Complications related to pregnancy, labor and delivery	1.2	13	Diseases of the nervous system	2.6	13	Nutritional deficiencies	2.0
14	Diseases of the digestive system	1.2	14	Tuberculosis	1.4	14	Intestinal infections	1.7
15	Injury and accidents	0.8	15	Whooping cough	1.4	15	Malaria	1.5
16	Diphtheria	0.4	16	Complications related to pregnancy, labor and delivery	0.9	16	Complications related to pregnancy, labor and delivery	0.6
			17	Malaria	0.4	17		
			18	Diphtheria	0.3	18		

### Trends in life expectancy and under-five mortality (1976–2012)

**Fig. 3:**
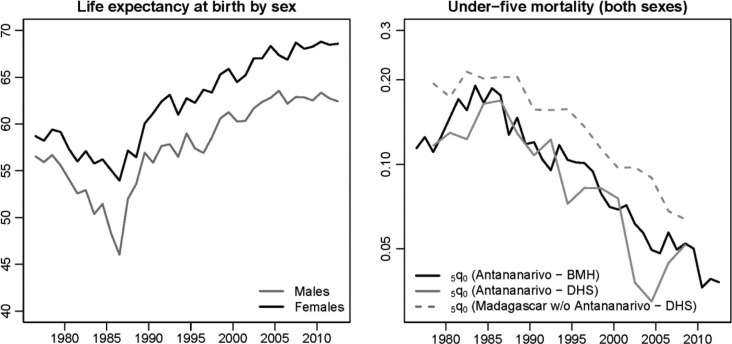
Trends in life expectancy at birth by sex and the under-five mortality rate (on a logarithmic scale) in Antananarivo city (1976–2012), according to data from the BMH and DHS.

In 1976, the life expectancy at birth had reached 57 years for males but it dropped to 46 in 1986 (Fig. 3). Over the same period, females lost 5 years of life expectancy, from 59 to 54. The under-five mortality rate, which was estimated at 114‰ in 1976, reached 190‰ in 1983. This peak is also evident in estimates from DHS, obtained by pooling together all birth histories of women living in the capital and interviewed in 1992, 1997, 2004 and 2009. It is worth noting that DHS estimates follow quite closely those of the BMH, in support of the hypothesis that the registration of deaths is almost complete in Antananarivo. Adults were also severely affected by this crisis; the probability of dying between ages 15 and 60 peaked at 0.51 among males in 1986, against 0.29 in the period 1976–1979.

Since 1986, Antananarivo has benefitted from an unabated decline in mortality. Life expectancy has risen steadily to reach 62.4 years for males and 68.6 for females in 2012. The under-five mortality rate has decreased five-fold from its level in 1983. The decline in mortality in the city has been slightly more rapid than observed in the rest of the country. In Fig. 3, estimates for the rest of the country are again obtained by pooling together the birth histories collected in the four DHS and excluding reports from mothers living in Antananarivo.

### Changes in cause-specific mortality rates

**Fig. 4:**
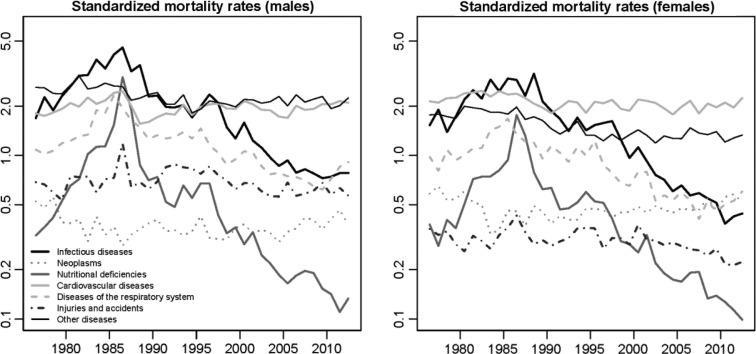
Standardized mortality rates for the main categories of causes of death (using the age structure of 1993 as a standard), based on data from the BMH.

Figure 4 presents, on a log scale, the standardized mortality rates for the main categories of causes of death (using the age structure of 1993 as a standard). The peak in 1984–1988 was predominantly due to a sheer increase in death rates from infectious diseases (malaria in particular) and nutritional deficiencies.

After 1986, the rapid decline in mortality from nutritional deficiencies is particularly salient. A negligible fraction of deaths are now due to these deficiencies. Infectious diseases still remain among the major killers, but have declined markedly, especially diseases targeted by the EPI. Diseases of the respiratory system have also declined since 1986 but to a lesser extent. By contrast, little progress has been made in the reduction of injuries, accidents and CVDs since the end of the 1980s.

**Fig. 5:**
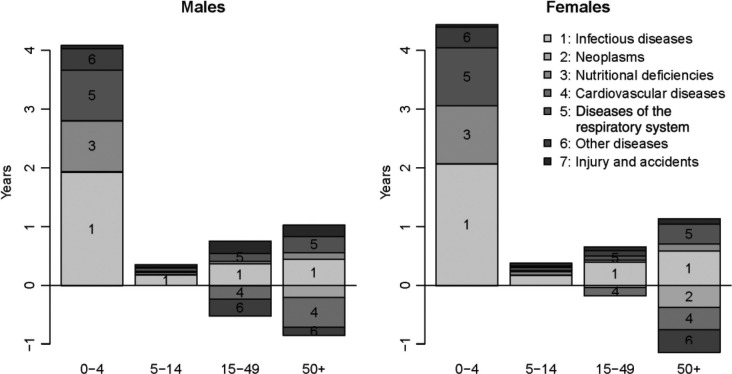
Contribution of the main categories of causes of deaths to changes in life expectancy (by sex) between 1990–1994 and 2008–2012 in Antananarivo.

In Fig. 5, we show the specific contribution of each category of causes in the change in life expectancy between two periods: 1990–1994 (when mortality had stabilized at levels observed prior to the crisis) and 2008–2012 (the past few years). For both sexes, the reduction of under-five mortality explains more than 80% of the gains in life expectancy, and about half of these gains in childhood are related to reductions in infectious diseases, the other half being mostly attributable to reductions in nutritional deficiencies and diseases of the respiratory system. Gains in life expectancy have been much more modest among the population aged 5 and over; reductions in infectious and respiratory diseases have been largely offset by increases in mortality from CVDs, neoplasms and other diseases, particularly at ages 50 and over.

## Discussion

Death registers of Antananarivo represent a valuable population-based mortality database for Madagascar, but they nonetheless suffer from important shortcomings. First, although deemed very high, the completeness of death reporting in the capital remains unknown and it certainly changed over time. For the period prior to 1960, there is no estimate of the completeness; we can only count on the fact that the health situation was more closely monitored in the capital than anywhere else because it was the seat of the General Government and had a large European population (16). After 1960, there are some signs that most deaths were captured in the vital registration system. In 1965, the CDR for the larger area of the province in which the city is located (10–11‰) was very close to the estimate based on the 1966 demographic survey (13–19‰). Virtually all infant deaths were reported in the province of Tananarive in 1965, compared to only 40% of infant deaths in the provinces of Diego-Suarez and Tulear (42). For the more recent period, we showed that trends in under-five mortality derived from death registers largely conform to those obtained from DHS. To go beyond these encouraging signs, however, it is necessary that we wait for the next census to conduct a proper analysis of the completeness of death reporting through standard demographic techniques (43).

A second limitation of death registers is related to the assignment and coding of causes of deaths. One must remain particularly circumspect in interpreting cause-of-death statistics of the start of the 20th century. Syphilis is a good example of this. It was considered by the French administration to be one of the main causes of depopulation in Madagascar, with an estimated prevalence of 60–75% in adults (44). In the monthly reports, it was singled out as accounting for as much as 9.5% of the reported deaths in 1901 (excluding stillbirths). This is probably exaggerated because of the difficulty in distinguishing between syphilis and what was called *farasisa* by midwives and native doctors, a catch-all term used to describe all chronic diseases with cutaneous symptoms (16). In 1902, the shortest list of the First Revision (ICD-1) of the International Classification of Diseases started to be used for the monthly reports and syphilis was subsumed into the ‘other causes’; altogether these accounted for only 3.7% of deaths.^1^ These variations highlight the need for caution and indicate that the figures presented here should be considered as broad indications rather than precise estimates. Even for the most recent period, the assignment of causes of deaths in hospital records or by the physicians of the BMH, and the coding which was done for this study, might be affected by errors.

Despite these limitations, death registers yield a coherent picture of the transition that took place in Antananarivo since 1900. Substantial changes in health and disease patterns occurred only from the 1940s. This decline coincided with the start of the indoor spraying of DDT and the introduction of chloroquine; the percentage of deaths attributable to malaria declined from 20% in 1946 to 5% in 1951 in the city (21). Further health improvements were achieved between 1951 and 1976, probably thanks to the diffusion of antibiotics. The chronology of the introduction of antibiotics and their impact on mortality trends awaits further study. However, the steep decline in the proportion of deaths due to pneumonia, bronchitis, influenza and tuberculosis between the years 1900–1903 and 1976–1980 indicates that they played a significant role. As for immunizations, the impact of the EPI was first obfuscated by the major mortality crisis of the mid-1980s but it became clearly apparent in the following decades. Not one death from measles has been reported to the BMH since 2005, even though measles accounted for 8% of deaths under age 5 in the period 1976–1980 and as much as 14% during the 1985 epidemic. Likewise, tetanus, polio, diphtheria and pertussis together caused 3% of under-five deaths in 1976–1980, and not one death from these four causes has been reported since 2005.

Gains in education undoubtedly contributed to the increased use of modern medical technology and the earlier recognition and treatment of diseases. By contrast, there is enough evidence to suggest that the mortality decline was not supported by rises in standards of living or improvements in nutrition. Within the capital city, it is estimated that the per capita consumption declined by 45% between 1961 and 1995, with a decline of 34% in family expenditures allocated to food and a decline of 44% in health care spending (32). Between the DHS conducted in 1992 and 2008/9, there has been little, if any progress in the reduction of stunting (growth retardation associated with chronic malnutrition). The percentage of children aged less than 5 years with moderate and severe stunting was 47.7% in the capital city in 1992, and 46.8% in 2008/9 (25, 28).

The experience of Antananarivo thus supports the view that changes in living standards and nutrition in developing countries were largely insufficient to explain the rapid gains in life expectancy after the World War II (45). The fact that the mortality decline was so dependent on anti-parasitic and anti-microbial medicine raises concerns in the context of increasing threads from drug-resistant infections (46, 47). It also bears implications for the future of the transition, as it means that the health care system has a crucial role to play despite persistent political turmoil and scarcity of public resources. From a theoretical perspective, the case of Antananarivo also raises a more general question, which is whether the likelihood of experiencing mortality reversals or stalls in the transition varies according to the type of factors that are driving the mortality declines (changes in income, nutrition, education or medical advances). Not only did the epidemiological transition start late in Antananarivo, it also proceeded by fits and starts, with important setbacks, particularly in the mid-1980s. In this sense, this transition bears some resemblance to the *protracted polarized model* conceptualized by Frenk et al. (48) and used recently to describe the changes occurring in Accra (8) and South Africa (9). In this model, counter-transitions can occur. Frenk et al. (48) also showed that the different stages of the epidemiological transition identified by Omran (1) are not necessarily sequential but can overlap for a considerable amount of time, resulting in the coexistence of infectious diseases and nutritional deficiencies with NCDs. In Antananarivo, it is only after 1990 that mortality rates from infectious diseases started to decline clearly below those attributable to CVDs. This relates to the notion of ‘double burden of disease’ experienced in many other African countries (49, 50). Another important dimension of this model, that we could not investigate here, is that transitions are thought to progress at varying speeds in the different socioeconomic groups, leading to a widening of inequalities in health between various segments of the population. It is likely that there has been substantial intra-urban variation in the pace of the transition in Antananarivo and this should be further explored. More attention should also be devoted to the mortality differentials between the capital city, other urban areas and the rest of the country. Other cities have a BMH in place, and some of them are known to maintain a virtually complete registration of deaths, such as Antsirabe, the third largest city of Madagascar (51). A comparative analysis could reveal whether an ‘epidemiological polarization’ is occurring across regions.

The diversity within SSA makes it difficult to generalize our findings to a broader context, especially because the HIV epidemic has dramatically distorted the epidemiological profile of several countries of mainland SSA. Madagascar has succeeded in keeping its HIV prevalence among the adult population below 1%, despite high rates of sexually transmitted infections such as syphilis. Mortality rates in adults and children have evolved in tandem. Progress in adult mortality has remained limited, however, because the decline in mortality rates caused by diseases predominant in the pre-transitional stage has been partly offset by the increase in mortality due to NCDs. Few data exist on risk factors for NCDs in Antananarivo. Obesity levels remain low in the city [3% of women aged 15–49 in 2008–2009 (28)], but prevalence of hypertension (blood pressure level of 140/90 mmHg or higher), estimated at 23% in 1997 and 28% in 2009 among the adult population (52, 53), is comparable to other urban areas in Africa. According to death registers, neoplasms account for a smaller proportion of deaths (7% in 2012), but standardized mortality rates from neoplasms have been on the rise over the past two decades, from 0.42 per thousand in 1990 to 0.61 in 2012.

Finally, this overview illustrates that death registration in major cities can provide valuable insights into the changes in patterns of mortality by cause. This had been previously demonstrated in other cities such as Abidjan (1986–1992), Saint-Louis (1930–1988), and Bamako (1974–1985) (54–56), but there has been little update since the 1990s. When high coverage is maintained, the registration of deaths offers a basis for monitoring the health system performance and evaluating the impact of interventions. In Madagascar in 2006, the Ministry of Health and Family Planning introduced the *National Health Sector Strategy and Development Plan* (PDSS) that expired in 2011 and was followed by an interim short-term plan. The performance of these plans could be reviewed based on data from the death registers, and new targets could be set for specific cause-specific mortality rates, beyond the focus on child mortality and on maternal deaths. In the future, achieving further health improvements will require a more effective fight against CVDs and increased efforts to reverse the upward trends observed in neoplasms, while at the same time addressing the unfinished agenda of communicable diseases.

## Conclusion

Our findings demonstrate that the epidemiological transition in Antananarivo has been protracted, with a pronounced mortality crisis in the mid-1980s followed by a very rapid decline in child mortality that was not supported by sustained economic growth or improved nutrition. Progress in adult survival have been more limited because gains in mortality due to infectious diseases and diseases of the respiratory system have been partly offset by increases in CVDs, neoplasms and other diseases. Antananarivo now clearly faces a double burden of communicable diseases and NCDs.

**Main findings**

- Epidemiological transition has been delayed in Antananarivo, Madagascar’s capital city. Mortality rates first declined after the 1940s and then resurged again in the mid-1980s, due to a temporary re-emergence of malaria and the collapse of Madagascar’s economy.
- Between 1986 and 2012, life expectancy at birth increased steadily from 54 to 69 among females and from 46 to 62 among males. Under-five mortality rates declined dramatically, despite persistent political instability and long-lasting economic crisis.
- Antananarivo now faces a double burden of disease. Cardiovascular diseases (CVDs), neoplasms and trauma account for more than 50% of deaths (compared to 9% around 1900). Infections and nutritional deficiencies account for approximately 12% of deaths, partly due to moderate and severe stunting in more than 40% of children under age 5.

**Key messages for action**

- Further gains in life expectancy require more effective interventions for CVDs and neoplasms, while concurrently addressing the unmet goals with regards to communicable diseases. Adult health needs require more attention, due to the limited progress in adult survival in recent years.
- Death registers maintained in major cities, though not able to compensate for the lack of a comprehensive vital registration system at the national level, could be further exploited to evaluate the performance of the health system and monitor intervention impacts in the urban population.
- Other capital cities in Sub-Saharan Africa have also set up a system of routine registration of deaths; however, the completeness of death reporting is often unknown and the data are rarely analysed. More research is required to evaluate whether such systems provide valuable causespecific mortality statistics.

## Footnotes

1 Prior to 1902, the diseases were probably classified according to the classification used in Paris and other French cities of more than 10,000 inhabitants starting in 1887.
